# A Rare Cause of Pain in a Patient Following Roux-en-Y Choledochojejunostomy

**DOI:** 10.1155/2024/5536057

**Published:** 2024-07-25

**Authors:** Kristiana Sather, Matthew Wheelwright, Guru Trikudanathan, Gregory Beilman

**Affiliations:** ^1^ Department of Surgery University of Minnesota, 420 Delaware Street SE, Mayo Mail Code 195, Minneapolis MN 55455, USA; ^2^ Department of Gastroenterology University of Minnesota, 420 Delaware Street SE, Mayo Mail Code 36, Minneapolis MN 55455, USA

## Abstract

**Introduction:**

Sump syndrome is a rare complication following bilioenteric anastomosis, most commonly following choledochoduodenostomy. This is only the third case in the literature of sump syndrome of the distal common bile duct (CBD) following end-to-side Roux-en-Y choledochojejunostomy (RYCJ). *Case Presentation*. A 69-year-old man with a history of end-to-side RYCJ for recurrent primary choledocholithiasis presented 3 years postoperatively with right upper quadrant (RUQ) abdominal pain affecting his quality of life. The work up revealed mild leukocytosis and computed tomography (CT) imaging that showed dilation of the distal CBD remnant. He underwent endoscopic retrograde cholangiopancreatography (ERCP) with the removal of large amounts of debris with initial resolution of his pain, but the pain recurred after several months and after two further ERCPs with only short-term resolution of pain, he eventually underwent an open distal CBD excision and the pain has since resolved.

**Conclusions:**

This case report describes a rare case of sump syndrome following RYCJ that presented with abdominal pain alone. Sump syndrome may have a wide array of presenting symptoms, and the pathophysiology of sump syndrome varies based on bilioenteric reconstruction. Although it has rarely been reported to occur in the distal blind CBD remnant following either RYCJ or hepaticojejunostomy, it is important to consider this in the differential for patients with abdominal pain following any bilioenteric reconstruction.

## 1. Introduction

For patients with recurrent primary choledocholithiasis who have undergone multiple endoscopic retrograde cholangiopancreatographies (ERCP), Roux-en-Y choledochojejunostomy (RYCJ) or hepaticojejunostomy (RYHJ) is often the next step in management. RYCJ and RYHC are reported to be as effective as choledochoduodenostomy (CDD), and though there is a similar incidence of overall complications between procedures, RYCJ and RYHC have been shown to have lower rates of recurrence, gastritis/reflux, and sump syndrome [1]. We present a rare cause of right upper quadrant (RUQ) abdominal pain in a patient who had previously undergone RYCJ. This case report is aligned with the CARE guidelines [2].

## 2. Case Presentation

A 69-year-old man with a history of laparoscopic cholecystectomy and multiple ERCPs for recurrent choledocholithiasis and cholangitis underwent an end-to-side RYCJ for recurrent primary choledocholithiasis. More than 1 year postprocedure, he developed occasional RUQ abdominal pain for which an abdominal ultrasound was obtained that showed no intra or extrahepatic biliary ductal dilation; however, the intrapancreatic CBD remnant was not well visualized. 3 years postoperatively he presented again to the clinic due to the persistence and increased frequency of RUQ abdominal pain. His exam was unremarkable, there was no hernia present or tenderness. Laboratory investigation demonstrated a WBC of 13.5, but normal alkaline phosphatase, ALT, AST, and bilirubin.

**Figure 1:**
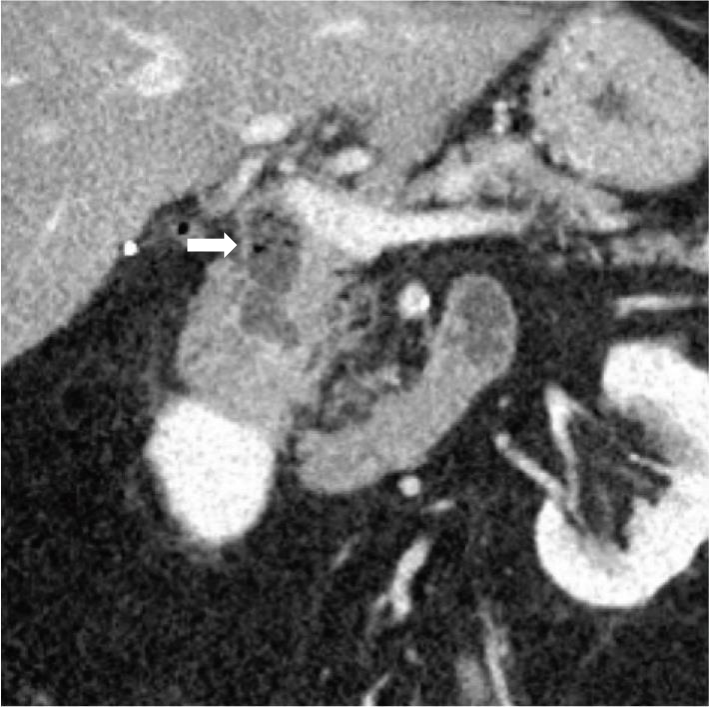
CT scan of the abdomen with arrow indicating dilated intrapancreatic CBD with accumulation of debris and stones, mimicking a duodenal diverticulum.

Computed tomography (CT) scan showed a 1.6 cm air and fluid-filled collection initially interpreted as a duodenal diverticulum. Upon review, this was felt to represent the intrapancreatic portion of the distal CBD (Figure 1).

**Figure 2:**
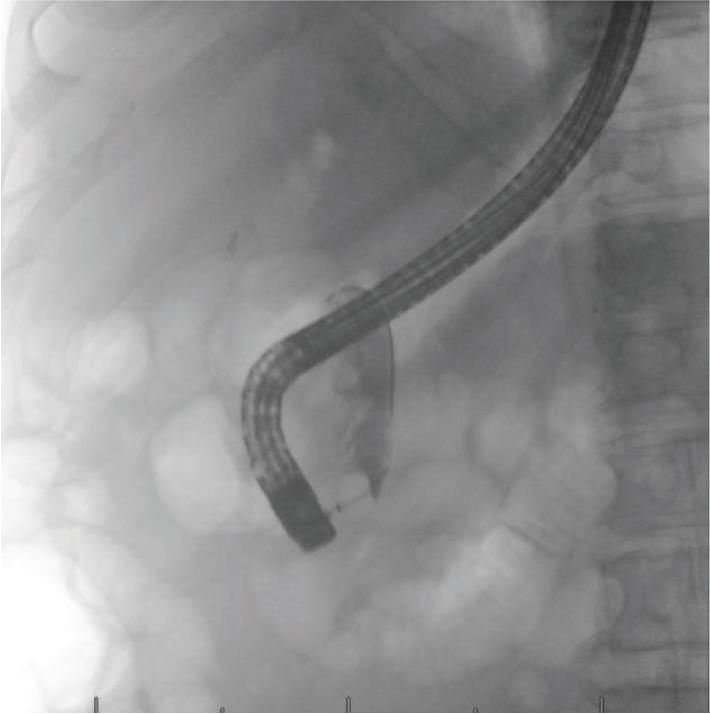
Cholangiogram showing dilated and partially obstructed distal CBD stump.

**Figure 3:**
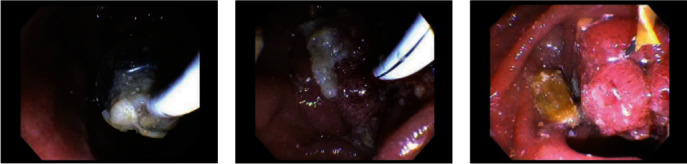
Balloon extraction of food material, sludge, and white stones. Noted by the advanced gastroenterologist to have a healthy appearing major papilla in the descending duodenum.

The patient underwent an outpatient ERCP with the successful removal of large amounts of food material, sludge, and white stones from the bile duct using a 15 mm balloon (Figures 2 and 3). During ERCP, the prior sphincterotomy was noted to be stenosed and a biliary sphincterotomy was performed. There was no duodenal diverticulum seen during ERCP. No further imaging such as endoscopic ultrasound or magnetic resonance cholangiopancreatography (MRCP) was obtained prior to ERCP due to the likely culprit of debris in the CBD stump seen on CT imaging, and the dual benefit of imaging and treatment that ERCP affords.

**Figure 4:**
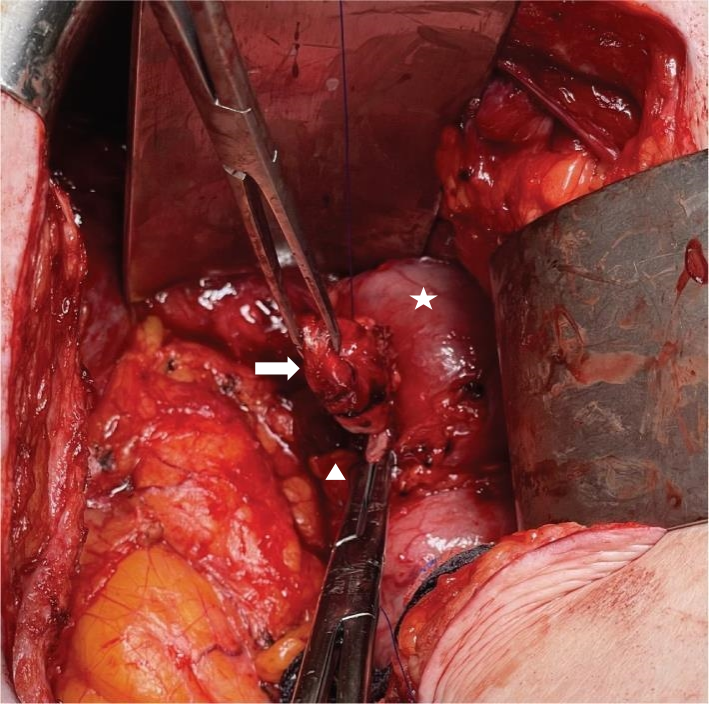
Isolated distal CBD remnant indicated by white arrow, Roux limb of prior choledochojejunostomy indicated by white star, and pancreas indicated by white triangle.

The patient initially had resolution of his symptoms, but after several months he developed recurrence of the RUQ pain and eventually underwent two further ERCPs with similar findings and only short-term resolution of his pain. Following the third ERCP, elective CBD resection was performed. Abdomen was entered via the prior right subcostal incision and the dilated (to 1.5 cm) distal CBD remnant was identified and isolated (Figure 4). This was dissected to the wall of the duodenum with minimal disruption of the uncinate process of the pancreas. CBD remnant was ligated (total length of 2.7 cm) and closed with 3–0 prolene. The operation lasted 2.5 hr, there were no intraoperative complications, estimated blood loss was 20 mL, a 19 Fr Blake drain was placed in the hepatic recess near the repair that was removed on postoperative day 3 following minimal drain output and a normal drain amylase, and he was discharged home on postoperative day 6. Final pathology was consistent with cholangitis, interestingly with infiltration of neutrophils, lymphocytes, and IgG4-positive plasma cells.

Postoperative course was complicated by low-grade fevers, chills, and a 5 cm fluid collection around the uncinate process seen on a CT scan at 1 month postoperatively. This was observed, his symptoms resolved, and on repeat CT scans, the fluid collection had resolved. His RUQ pain improved significantly following surgery and eventually resolved for approximately 9 months until he again developed RUQ abdominal pain. This was different in nature from the prior, attributed to small intestinal bacterial overgrowth, and resolved with a course of antibiotics. At this point, he was pain free.

## 3. Discussion

RYHJ and RYCJ are considered the standard method of biliary reconstruction and are most commonly performed to repair benign or iatrogenic biliary strictures, or for benign or malignant CBD obstruction [3, 4, 5]. When looking at all indications (benign or malignant), morbidity and mortality rates range from 26% to 42% and 2% to 6.5%, respectively [5]. The most common long-term complications include recurrent cholangitis and anastomotic stricture formation, followed by secondary sclerosing cholangitis, incisional hernia, duodenal ulcer, and adhesive bowel obstruction [6, 7, 8]. These complications often presents with abdominal pain and/or laboratory abnormalities. Reflux or gastritis is more common following CDD and is often a reason for conversion to RYCJ or RYHJ [9, 10]. Another cause of abdominal pain for patients who have undergone RYHJ or RYCJ, particularly when done for primary choledocholithiasis, is recurrence of CBD or intrahepatic stones, which can occur at a rate of 0%–14.3% [9, 11].

A rare complication of bilioenteric anastomosis, including RYHJ and RYCJ, is sump syndrome, which can manifest in a variety of presentations, including abdominal pain, jaundice, pancreatitis, and cholangitis [1]. The 1%–5% risk (and up to 9.6% in some studies) of sump syndrome following CDD is one reason why RYHJ and RYCJ are the preferred operations for patients requiring biliary reconstruction [1, 12]. Given that RYHJ or RYCJ are now more commonly performed, sump syndrome is now more frequently being described as a complication of these operations. Although the rate of sump syndrome after RYHC/RYCJ is not well established, there are several case series and studies that have described this phenomenon.

The pathophysiology of sump syndrome varies based on surgical technique, but in general, the CBD serves as a reservoir, or “sump,” for debris and stone formation. For side-to-side CDD, the CBD distal to the anastomosis acts as the sump. For end-to-side CDD, the reflux of enteric contents retrograde through the anastomosis and into the CBD leads to the accumulation of debris. The most commonly described pathophysiology of sump syndrome following RYHJ is the reflux of enteric contents retrograde into the hepatic ducts from the Roux limb [1, 3]. This has usually been attributed to an inadequate length of the Roux limb (<50 cm) or an alteration in intestinal motility [3, 13, 14]. There are two reported cases of sump syndrome in the distal CBD remnant [15, 16]. Robbins et al. [16] describe a patient with primary sclerosing cholangitis who underwent a liver transplant with RYHJ reconstruction where the distal CBD remnant had accumulated debris, and due to the loss of peristalsis and filling pressures, had become a sump. This is similar to our patient and resolved with sphincterotomy and dilation of distal CBD stenosis. The other case, described by Eshkanazy et al. [15], was due to a fistula between the distal CBD remnant and the jejunum. In an effort to address these risks while avoiding unnecessary dissection of the CBD, some authors propose either occlusion or resection of the distal CBD in the setting of a patulous papilla (due to the increased risk of retrograde reflux), or resection of the intrapancreatic distal CBD only if it is significantly dilated [17]. In our patient, at the index operation, the distal CBD was irrigated with sterile saline until clear and then closed with a 2–0 Vicryl stitch in a running fashion. On reoperation, the distal CBD was closed with a 3–0 Prolene stitch in a running fashion.

Amongst patients with sump syndrome, the presentation can be variable and range from vague abdominal pain, as was the case in our patient, to recurrent cholangitis, liver abscess, and recurrent pancreatitis [3, 12, 18]. In a case series of 30 patients with sump syndrome following side-to-side CDD, the majority of patients presented with abdominal pain and fever and had abnormal liver function tests, whereas only 16% presented with abdominal pain alone [12]. Other than a mild leukocytosis, our patient had normal labs, further obfuscating the diagnosis. Although this was his first presentation with regard to abdominal pain, it had been going on for at least a year, which is similar to the average of 1.5 years between the onset of symptoms and the diagnosis previously reported [18]. Even though our patient was not ill, his abdominal pain was affecting his quality of life, and it was beneficial for the patient to obtain imaging early on in his presentation. Because of the variability in presentation and the potential risk of more serious manifestations, including cholangitis and pancreatitis, it is important to consider this rare diagnosis in the differential for any patient with biliary reconstruction.

**Figure 5:**
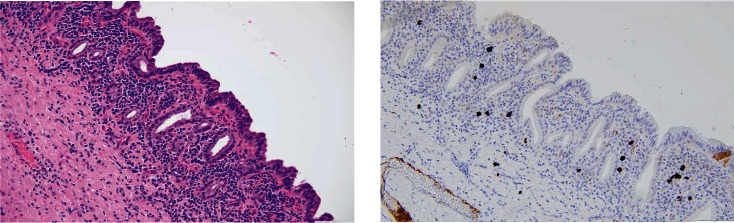
H and E stain showing infiltration of inflammatory cells, and IgG4 stain showing clusters of IgG4-positive plasma cells.

Interestingly, the final pathology showed a neutrophilic and lymphoplasmacytic infiltration of the epithelium, and IgG4 immunohistochemical stain showed IgG4 positive plasma cells numbering up to 25/high powered field (hpf; Figure 5). This is likely due to chronic inflammation due to recurrent sump syndrome rather than due to IgG4-related disease (IgG4-RD) as there were no characteristic sclerotic changes of the bile duct, he has had no other clinical manifestations of autoimmune pancreatitis or extrapancreatic IgG4-RD, and does not meet the histologic diagnostic scheme of IgG4-RD per the Boston criteria [19, 20]. He only meets 1/3 of the characteristic histologic features and has <50 IgG4-positive plasma cells/hpf of the surgical specimen [20]. However, given the diversity of clinical manifestations and varying effects on multiple organ systems that IgG4-RD can have, if he were to develop recurrent RUQ abdominal pain not attributable to other causes or other concerning findings, this would be considered in the differential.

## 4. Conclusion

We present a case of sump syndrome of the distal CBD remnant as a rare cause of abdominal pain in a patient who had previously undergone RYCJ. More commonly considered in the setting of cholangitis or pancreatitis, and following CDD, it is important to consider sump syndrome in a patient whose only symptom is abdominal pain and has previously undergone RYCJ. We would thus recommend at least considering distal CBD excision during the primary operation in an attempt to minimize the rare but possible complication of sump syndrome.

## Data Availability

The sump syndrome and outcomes data supporting this case report are from previously reported studies and datasets, which have been cited. The processed data are available from the corresponding author upon request.
